# An Efficacy Predictive Method for Diabetic Ulcers Based on Higher-Order Markov Chain-Set Pair Analysis

**DOI:** 10.1155/2020/5091671

**Published:** 2020-06-16

**Authors:** Le Kuai, Xiao-ya Fei, Jia-qi Xing, Jing-ting Zhang, Ke-qin Zhao, Kan Ze, Xin Li, Bin Li

**Affiliations:** ^1^Department of Dermatology of Yueyang Hospital, Shanghai University of TCM, Shanghai 200437, China; ^2^Institute of Dermatology, Shanghai Traditional Chinese Medicine Research Institute, Shanghai 201203, China; ^3^Department of Japanese, Osaka College of High Technology, Osaka 532-0003, Japan; ^4^Department of Ophthalmology, Shanghai General Hospital (Shanghai First People's Hospital), Shanghai Jiao Tong University, School of Medicine, Shanghai 200080, China; ^5^Zhuji Research Institute of Connection Mathematics, Zhuji 311811, China; ^6^Department of Dermatology, Shaanxi Traditional Chinese Medicine Hospital, Xi'an 710003, China

## Abstract

**Background:**

Clinical comprehensive decision-making of diabetic ulcers includes curative effect evaluation and curative effect prediction. Nevertheless, there are few studies on the prediction of diabetic ulcers.

**Methods:**

Set pair analysis (SPA) was used to assess the curative effect evaluation, and therapeutic effect was evaluated by connection degree (CD). The higher-order Markov chain-SPA curative effect prediction model was established to predict the future curative effect development. The predicted results with higher-order Markov chain-SPA and traditional first-order Markov-SPA model were compared with the actual results of the patients to verify the effectiveness of prediction.

**Results:**

The connection degree of index levels I and II of 15 patients with diabetic ulcers after traditional Chinese medicine (TCM) treatment increased with time, while that of index levels IV and V decreased, indicating that the curative effect tends to improve. The higher-order Markov chain-SPA model was used to predict the curative effect. The results showed that the relative errors were fewer than the traditional first-order Markov-SPA model.

**Conclusions:**

The present study suggests that a method of SPA combined with higher-order Markov-SPA is relatively effective and can be applied to the clinical prediction of diabetic ulcers, which has higher accuracy than traditional first-order curative effect prediction model.

## 1. Introduction

Medical decision-making has always been the core and key issue of clinical medicine. The comprehensive decision-making includes not only evaluation of the current symptoms or indicators of patients but also prediction research of future therapeutic effects. At present, there are many methods for medical comprehensive decision-making. Wang et al. [1] evaluated medical quality by a dynamic technique for order performance by similarity to ideal solution. Xu et al. [2] used random forest and information gain algorithm to establish a syndrome classification model that accords with the dialectical theory of TCM. Nevertheless, research on the prediction of curative effect has not been paid enough attention at present.

By the end of 2017, it was estimated that there are 451 million (age: 18–99 years) people with diabetes worldwide. Furthermore, these figures were expected to increase to 693 million by 2045 [3]. Diabetic ulcer is a common serious complication of diabetes and also the main cause of disability in patients [4]. Recently, TCM has become a complementary and alternative medicine worldwide and has been gradually used in the treatment of diabetic ulcers. Our prestudy has reported that Sheng-ji Hua-yu (SJHY) treatment is a sequential therapy method that is efficient in the management of diabetic skin ulcers, making their wound-healing time 2-3 days less than conventional western medicine group [5, 6]. Mechanism of the action might be related to the inhibition of Activin/Follistatin [7].

With respect to curative effect evaluation, Cloud Model-Set Pair Analysis (CM-SPA) is demonstrated to an efficacy assessment for diabetic ulcers with SJHY treatment [8]. On the basis, it is therefore significant to explore a curative effect prediction model. Markov chain is designed to describe dynamic random phenomena that have the discrepancy of time and state. In the prediction of clinical efficacy, a prediction model must be chosen which can handle the transition probability between the states of each treatment time point. What is more, the basic method of Markov chain prediction is to use the transition probability matrix between states to predict the state of events and its development trend [9]. SPA theory is brought up to make a comprehensive analysis of certain and uncertain information. Its core idea is to construct a set pair for two sets associated with uncertain systems to analyze the identity, discrepancy, and contradistinction. Then CD of set pair is defined to quantitatively describe uncertainties caused by ambiguity, randomness, and incomplete information [10]. Clinical efficacy evaluation is an uncertain model caused by individual differences, environmental factors, and other factors, and the uncertainty will convert accordingly with the change of state.

The SPA-Markov method has been used to construct the prediction model of clinical curative effect. This method is composed of SPA and Markov chain that analyze and predict uncertain systems with the characteristics of dynamics, continuity, and randomness. It has been widely used in the fields of aviation safety dynamic assessment [11], information security risk assessment [12], gas pipeline hazard prediction [13], hydropower plant production environmental safety behavior evolution prediction [14], and so on. In addition, Markov model has many developments, including higher-order Markov model [15], multivariate Markov model [16], and hidden Markov chain model [17]. According to the research report, the traditional first-order Markov chain assumes that the next future probability structure is only related to the current state and does not account for its history, consistent with the First-order non-aftereffect. However, in clinical practice, the development of symptoms of patients is in constant metamorphosis that is not completely determined by the recent state but is related to a period of treatment. While the traditional first-order Markov chain has found many applications in prediction model, it abandons long-term useful information when describing random phenomena and is relatively rough in the description of the development and change process of the system that is easy to lead to the distortion of prediction results in practical application. In order to improve the Markov model closer to the real situation and get better prediction results, Raftery [18] firstly proposed the concept of higher-order Markov model to extend the traditional first-order correlation to higher-order correlation, which includes the more preliminary information into forecasts of future variables and the more precise random prediction so that it improves the accuracy of prediction results [19].

**Figure 1:**
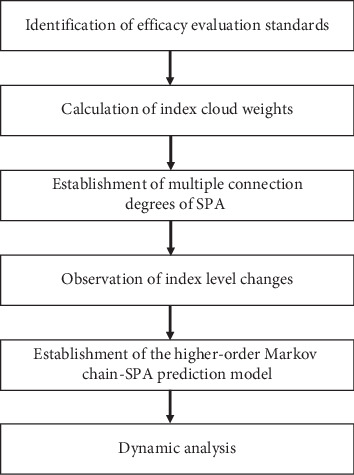
The process of curative effect evaluation and prediction. The process of curative effect evaluation and prediction was carried out by using the higher-order Markov chain-SPA model.

In this paper, a SPA model based on higher-order Markov chain was applied to efficacy evaluation and prediction of 15 patients with diabetic ulcers during TCM treatment. According to the following evaluation and prediction process (Figure 1), the SPA method was used to establish the efficacy evaluation model to evaluate the therapeutic effect. Then the higher-order Markov-SPA prediction model was established to predict the future development of curative effect. The predicted results with higher-order Markov chain-SPA and traditional first-order Markov-SPA models were compared with the actual results of the patients to reveal the effectiveness of prediction.

## 2. Methods

### 2.1. SPA

SPA is one of the contact mathematics methods proposed by Zhao and Xuan [10], which deals with uncertain state and trend of the system in many practical problems. “Identity,” “discrepancy,” and “contradistinction” are used to describe their relations to each other and constructed into a certain-uncertain system.

In a given system, two sets *A* and *B* with certain relations form a set pair *H* (*A*, *B*). Assuming that the set has *N* characteristics, where *S* is the number of features shared in the set pair, *P* is the opposite number of features, and *F* is the number of uncertain features; *S* + *P* + *F* = *N*; the CD can be obtained as follows:

(1) $$\begin{array}{c} \mu =\frac{S}{N}+\frac{F}{N}i+\frac{P}{N},j=a+bi+cj. \end{array}$$

**Definition 1 .**

 *a*=*S*/*N*, *b*=*F*/*N*, *c*=*p*/*N* represents the degree of “identity,” “discrepancy,” and “contradistinction,” respectively, *a*+*b*+*c*=1, *i* denotes the uncertainty coefficient, *i* ∈ [−1,1], and *j* is the coefficient of opposites, which is generally defined as −1. Then connection could be expressed as

(2) $$\begin{array}{c} \mu =a+b_{1}i_{1}+b_{2}i_{2}+\cdots +b_{n}i_{n}+cj, \end{array}$$

and when *n* = 3, the CD of the five-element connection number is defined as

(3) $$\begin{array}{c} \mu =a+b_{1}i_{1}+b_{2}i_{2}+b_{3}i_{3}+cj=a+bi+cj+dk+el. \end{array}$$

Each symptom index has a different weight in the evaluation of curative effect; the weight of cloud model (CM) was used [8]. The specific calculation method of the symptom weight was given in the literature [20]. The standardized values of each index were obtained after CM characteristic numbers and the cloud weight calculation. Ex, En, and He are the CM characteristic numbers corresponding to each symptom index *k*. Ex is the expected value of the cloud drop that can represent the qualitative concept. Then, the weight value of symptom index *k* is defined as *ω*_*k*_.

**Definition 2 .**

Assume that, at time *t*, the numbers of symptoms in the five levels are *A* (*t*), *B* (*t*), *C* (*t*), *D* (*t*), *E* (*t*), and *A* (*t*) + B (*t*) + *C* (*t*) + *D* (*t*) + *E* (*t*) = *N*. The original *N* symptoms are reordered and numbered sequentially in the order of *A* (*t*), *B* (*t*), *C* (*t*), *D* (*t*), *E* (*t*). The weight corresponding to the serial number of each symptom is *ω*_*k*_(*t*) at time *t*.

According to equation (3), the CD of five-element connection number where symptom weight has been taken into consideration is calculated:

(4) $$\begin{array}{c} \mu \left(t\right)=a\left(t\right)+b\left(t\right)i+c\left(t\right)j+d\left(t\right)k+e\left(t\right)l=\sum_{k=1}^{A\left(t\right)}\omega_{k}\left(t\right)+\sum_{k=A\left(t\right)+1}^{A\left(t\right)+B\left(t\right)}\omega_{k}\left(t\right)i+\sum_{k=A\left(t\right)+B\left(t\right)+1}^{A\left(t\right)+B\left(t\right)+C\left(t\right)}\omega_{k}\left(t\right)j+\sum_{k=A\left(t\right)+B\left(t\right)+C\left(t\right)+1}^{A\left(t\right)+B\left(t\right)+C\left(t\right)+D\left(t\right)}\omega_{k}\left(t\right)k+\sum_{k=A\left(t\right)+B\left(t\right)+C\left(t\right)+D\left(t\right)+1}^{A\left(t\right)+B\left(t\right)+C\left(t\right)+D\left(t\right)+E\left(t\right)}\omega_{k}\left(t\right)l, \end{array}$$

where 0 ≤ *ω*_*k*_(*t*) ≤ 1,  ∑_*k*=1_^*N*^*ω*_*k*_(*t*)=1.

**Definition 3 .**

The five symptom index levels I to V were given the scores 9, 7, 5, 3, and 1 (Table 1). Afterwards, the efficacy score could be defined as

(5) $$\begin{array}{c} U=9a\left(t\right)+7b\left(t\right)+5c\left(t\right)+3\;d\left(t\right)+1e\left(t\right). \end{array}$$

Generally speaking, when score of curative effect is high, curative effect will be better.

### 2.2. Higher-Order Markov Chain

Markov chain was first proposed by Russian mathematician A. A. Markov in 1907. The purpose of the method is to describe dynamic random phenomena and it has been applied successfully in many fields of time series analysis and prediction. However, the traditional first-order Markov model only considers that the future probability structure is related to the current state and abandons the older useful information in describing random phenomena which course the process development roughly. In order to make the Markov chain closer to the real situation, Raftery and Tavare [21] first proposed the concept of higher-order Markov chain and pointed out that the traditional first-order correlation can be extended to higher-order correlation. If the time dynamic variable is only related to its previous continuous *n* states, it has nothing to do with *n* previous states, so this characterization is called *n*-order non-aftereffect. In the following series of papers, Raftery proposed the maximum likelihood method of mixed transfer distribution (MTD) model. The parameter estimation of the higher-order Markov model is studied and the transformation of the higher-order Markov model from theoretical results to practical application tools is realized. Based on the research of Raftery, Ching [22] extended the parameter limitation of higher-order Markov chain, deduced a more mature higher-order Markov model that is closer to objective reality, and discussed how to realize its parameter estimation by optimization method in 2004. The higher-order Markov chain method can be used to deal with more complex practical problems through these improvements.

The stochastic process {*C*(1),…, *C*(2),…, *C*(*T*)} is defined as Markov chain of discrete parameters, the state space Ω={1,2,…, *m*}, and *C*(*t*)=[*c*_1_(*t*), *c*_2_(*t*),…, *c*_*m*_(*t*)] denotes the probability distribution vector of each state at time *t*. For a positive integer *n*, here we have

(6) $$\begin{array}{c} C\left(t+1\right)=\sum_{r=1}^{n}\lambda_{r}C\left(t+1-r\right)Q_{r}. \end{array}$$

Then, {*C*(1),…, *C*(2),…, *C*(*T*)} is an n-order Markov chain, where *λ*_*r*_ ≥ 0 is a high-order coefficient and ∑_*r*=1_^*n*^*λ*_*r*_=1. The *M*-order square matrix *Q*_*r*_ can be considered as *r*-step state transition probability matrix.

It should be pointed that higher-order Markov chain describes the distribution of state *C* (*t* + 1) at time *t* + 1, which is related to the previous *n* time states *C* (*t*), *C* (*t* − 1), and *C* (*t* + 1 − *n*) and ignores the more previous states. The higher-order Markov chain extends the restriction of only adjacent dependence in the first-order Markov chain that makes the model more realistic.

### 2.3. Higher-Order Markov Chain-SPA Prediction Model

**Definition 4 .**

The number of indexes of curative effect level I is *A* (*t*) at time *t*. At time *t* + 1, there are *A* (*t*1) symptoms, the status of which is still level I, *A* (*t*2) symptoms change from level I to level II, *A* (*t*3) symptoms change from level I to level III, *A* (*t*4) symptoms change from level I to level IV, and *A* (*t*5) symptoms change from level I to level V. The state transition probability of the symptom index of level *I* during time [*t*, *t* + 1] was

(7) $$\begin{array}{c} Q_{A}=\left(\begin{array}{cc} p_{11} & \begin{array}{cc} p_{12} & \begin{array}{cc} p_{13} & \begin{array}{cc} p_{14} & p_{15} \end{array} \end{array} \end{array} \end{array}\right), \end{array}$$

where *p*_11_=∑_*k*=1_^*A*(*t*1)^*ω*_*k*_(*t*)/∑_*k*=1_^*A*(*t*)^*ω*_*k*_(*t*); *p*_12_=∑_*k*=*A*(*t*1)+1_^*A*(*t*1)+*A*(*t*2)^*ω*_*k*_(*t*)/∑_*k*=1_^*A*(*t*)^*ω*_*k*_(*t*); *p*_13_=∑_*k*=*A*(*t*1)+*A*(*t*2)+1_^*A*(*t*1)+*A*(*t*2)+*A*(*t*3)^*ω*_*k*_(*t*)/∑_*k*=1_^*A*(*t*)^*ω*_*k*_(*t*); *p*_14_=∑_*k*=*A*(*t*1)+*A*(*t*2)+*A*(*t*3)+1_^*A*(*t*1)+*A*(*t*2)+*A*(*t*3)+*A*(*t*4)^*ω*_*k*_(*t*)/∑_*k*=1_^*A*(*t*)^*ω*_*k*_(*t*); and *p*_15_=∑_*k*=*A*(*t*1)+*A*(*t*2)+*A*(*t*3)+*A*(*t*4)+1_^*A*(*t*1)+*A*(*t*2)+*A*(*t*3)+*A*(*t*4)+*A*(*t*5)^*ω*_*k*_(*t*)/∑_*k*=1_^*A*(*t*)^*ω*_*k*_(*t*).

Similarly, the state transition probabilities of other symptom indicators during time [*t*, *t* + 1] are *Q*_*B*_, *Q*_*C*_, *Q*_*D*_, and *Q*_*E*_, respectively, while the transition probability matrix *Q* (*t* + 1) of the system in [*t*, *t* + 1] is

(8) $$\begin{array}{c} Q\left(t+1\right)=\left[\begin{array}{c} \begin{array}{cc} p_{11} & \begin{array}{cc} p_{12} & \begin{array}{cc} p_{13} & \begin{array}{cc} p_{14} & p_{15} \end{array} \end{array} \end{array} \end{array}\\ \begin{array}{cc} p_{21} & \begin{array}{cc} p_{22} & \begin{array}{cc} p_{23} & \begin{array}{cc} p_{24} & p_{25} \end{array} \end{array} \end{array} \end{array}\\ \begin{array}{cc} p_{31} & \begin{array}{cc} p_{32} & \begin{array}{cc} p_{33} & \begin{array}{cc} p_{34} & p_{35} \end{array} \end{array} \end{array} \end{array}\\ \begin{array}{cc} p_{41} & \begin{array}{cc} p_{42} & \begin{array}{cc} p_{43} & \begin{array}{cc} p_{44} & p_{45} \end{array} \end{array} \end{array} \end{array}\\ \begin{array}{cc} p_{51} & \begin{array}{cc} p_{52} & \begin{array}{cc} p_{53} & \begin{array}{cc} p_{54} & p_{55} \end{array} \end{array} \end{array} \end{array} \end{array}\right]. \end{array}$$

The higher-order coefficient *λ* (*t* + 1 − *r*) is equivalent to the weight of the state transition probability matrix *Q* (*t* + 1 − *r*). If the state transition probability matrix *Q* (*t* + 1 − *r*) at time *t *+* *1 − *r* is more similar to the state transition probability matrix at other times, the contribution of the matrix is smaller and the higher-order coefficient *λ* (*t* + 1 − *r*) is smaller. Therefore, the higher-order coefficient calculation method can be constructed by using matrix similarity [23].

**Definition 5 .**

The state transition probability matrices of *t *+* *1* *−* r* and *t* +* *1* *−* s* at any two times are *Q* (*t *+* *1* *−* r*) and *Q* (*t* + 1 − *s*), respectively. The similarity is

(9) $$\begin{array}{c} \Theta_{\left(t+1-r,\;t+1-s\right)}=\cos\;\theta =\frac{\langle Q\left(t+1-r\right),\;Q\left(t+1-s\right)\rangle }{\left\|Q\left(t+1-r\right)\right\|\left\|Q\left(t+1-s\right)\right\|}, \end{array}$$

where <*Q* (*t* + 1 − *r*), *Q* (*t* + 1 − *s*)> = *tr* (*Q* (*t* + 1 − *s*)^T^*Q* (*t* + 1 − *r*)), *tr*(·) represents the sum of diagonal elements of a matrix, and ‖·‖ reflects the norm derived from the inner product of a matrix, which is

(10) $$\begin{array}{c} \left\|Q\left(t+1-r\right)\right\|=\sqrt{\langle Q\left(t+1-r\right),\;Q\left(t+1-s\right)\rangle }, \end{array}$$

(11) $$\begin{array}{c} \left\|Q\left(t+1-s\right)\right\|=\sqrt{\langle Q\left(t+1-s\right),\;Q\left(t+1-r\right)\rangle }. \end{array}$$

*θ* is the angle between the two matrices. When *θ* = 90° and Θ = 0, it is indicated that the two matrices are not similar; conversely, if *θ* = 0° and Θ = 1, the similarity of the two matrices is high. The more similar the state transition probability matrix is, the smaller the higher-order coefficient *λ* is defined. Therefore, the similarity matrix of the pairwise matrix between the matrices *Q* (*t*), *Q* (*t* − 1), and *Q* (*t* + 1 − *n*) is shown:

(12) $$\begin{array}{c} \Theta =\left[\begin{array}{cccc} 1 & \Theta_{t,t-1} & \cdots & \Theta_{t,t+1-n}\\ \Theta_{t-1,t} & 1 & \cdots & \Theta_{t-1,t+1-n}\\ \vdots & \vdots & \ddots & \vdots \\ \Theta_{t+1-n,t} & \Theta_{t+1-n,t-1} & \cdots & 1 \end{array}\right]. \end{array}$$

According to the similarity matrix Θ, the similarity between the matrix *Q* (*t* + 1 − *r*) and other matrices can be defined as

(13) $$\begin{array}{c} \gamma_{t+1-r}=\frac{1}{n}\sum_{r=1,r\neq s}^{n}\Theta_{t+1-r,\;t+1-s}. \end{array}$$

Furthermore, the high-order coefficient is calculated:

(14) $$\begin{array}{c} \lambda \left(t+1-r\right)=\frac{1-\gamma_{t+1-r}}{\sum_{r=1}^{n}\left(1-\gamma_{t+1-r}\right)}. \end{array}$$

**Definition 6 .**

The curative effect CD of *n* moments before time *t* is defined as follows:

(15) $$\begin{array}{c} \left\{\begin{array}{c} \mu \left(t\right)=a\left(t\right)+b\left(t\right)i+c\left(t\right)j+d\left(t\right)k+e\left(t\right)l,\\ \mu \left(t-1\right)=a\left(t-1\right)+b\left(t-1\right)i+c\left(t-1\right)j+d\left(t-1\right)k+e\left(t-1\right)l,\\ \vdots \\ \mu \left(t+1-r\right)=a\left(t+1-r\right)+b\left(t+1-r\right)i+c\left(t+1-r\right)j+d\left(t+1-r\right)k+e\left(t+1-r\right)l,\\ \vdots \\ \mu \left(t+1-r\right)=a\left(t+1-r\right)+b\left(t+1-r\right)i+c\left(t+1-r\right)j+d\left(t+1-r\right)k+e\left(t+1-r\right)l. \end{array}\right. \end{array}$$

CD of curative effect at time *t* + 1 can be predicted according to equation:

(16) $$\begin{array}{c} \hat{\mu }\left(t+1\right)=\hat{a}\left(t+1\right)+\hat{b}\left(t+1\right)i+\hat{c}\left(t+1\right)j+\hat{d}\left(t+1\right)k+\hat{e}\left(t+1\right)l=\sum_{r=1}^{n}\left[\lambda \left(t+1-r\right)\mu \left(t+1-r\right)Q\left(t+1-r\right)\right]{\left[1,i,j,k,l\right]}^{\text{T}}, \end{array}$$

where *t* = 1, 2,…, *T*; *Q* (*t* + 1 − *r*) is the transition probability matrix at time *t* + 1 − *r*; *λ* (*t* ≤ 1 − *r*) is the higher-order coefficient, and ∑_*r*=1_^*n*^*λ*(*t*+1 − *r*)=1.

### 2.4. Case Analysis

According to the previous clinical observation, ten indexes were identified as the main indicators that affect prognosis of diabetic ulcers [8, 24]. Each course of treatment lasted seven to fourteen days that a total of four treatment time points were observed. Referring to the previous literature of SPA model [25] and Markov model [26], it was found that four examples and two examples were used to verify the applicability of the model, respectively. Therefore, 15 patients were included in this research. Subsequently, the curative effects of 15 patients of diabetic ulcers treated with SJHY method were evaluated and predicted by higher-order Markov chain-SPA and first-order Markov chain-SPA, respectively. Considering the clinical circumstances of amelioration of patients' symptoms, the results were standardized in case the CD was not equal to 1.

Ten experts were invited to assess the importance of symptom indexes of diabetic ulcers (Table 2); then, the qualitative language variables were transformed into quantitative values by CM. SPA was set as a five-element model that represented five therapeutic levels of asymptomatic (I), lighter (II), moderate (III), heavier (IV), and severe (V), respectively. According to the IWGDF/IDSA classification [27] and referring to literature of diabetic foot ulcers [28], the evaluation of each therapeutic level (Table 3) was determined and the curative effect level (Table 4) of each patient after each course of treatment was obtained. According to the results, the small sample data were compared using the *t*-test or the Wilcoxon rank-sum test, as appropriate. Two-tailed *p* values < 0.05 were considered statistically significant. All calculations were carried out by SPSS (version 21.0) software package.

## 3. Results

**Figure 2:**
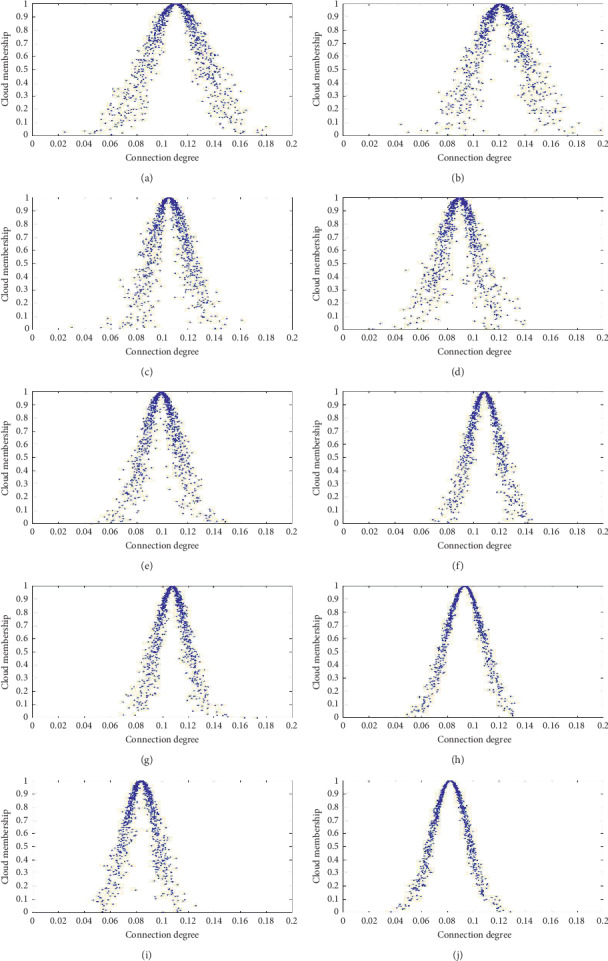
Cloud images of the index weight. (a–j) The cloud images corresponding to ten symptoms. The confusion value of each symptom is less than 1, which is not “fog,” indicating that the evaluation of symptom weight had practical value.

The value of cloud weight (Table 5) corresponding to each symptom index *k* was calculated by the cloud model and tested by the confusion degree test, where the confusion value is less than 1. The corresponding image of each symptom was cloud instead of fog (Figure 2) which could be used as the weight of the curative effect index.

The CD of 15 patients at each time (Table 6) could be calculated by equation (4) and the curative efficacy score *U* (Table 7) could be calculated by equation (5). Because the fourth cycle is predicted by the curative effect of the first three cycles, the higher-order coefficient is set as three. The state transition matrix and higher-order coefficient of each treatment course of 15 patients are calculated by equations (7)–(14).

The state transfer matrix of a 68-year male patient (patient a) in each treatment course was defined as follows:

(17) $$\begin{array}{c} Q\left(t1\right)=\left[\begin{array}{ccccc} 0 & 0 & 0 & 0 & 0\\ 0 & 1 & 0 & 0 & 0\\ 0.62 & 0.38 & 0 & 0 & 0\\ 0 & 0.35 & 0.65 & 0 & 0\\ 0 & 0 & 0 & 1 & 0 \end{array}\right],\\ Q\left(t2\right)=\left[\begin{array}{ccccc} 1 & 0 & 0 & 0 & 0\\ 0.64 & 0.36 & 0 & 0 & 0\\ 0 & 1 & 0 & 0 & 0\\ 0 & 0.30 & 0.70 & 1 & 0\\ 0 & 0 & 0 & 0 & 0 \end{array}\right],\\ Q\left(t3\right)=\left[\begin{array}{ccccc} 1 & 0 & 0 & 0 & 0\\ 0.55 & 0.45 & 0 & 0 & 0\\ 0 & 1 & 0 & 0 & 0\\ 0 & 0 & 0 & 0 & 0\\ 0 & 0 & 0 & 0 & 0 \end{array}\right]. \end{array}$$

His higher-order coefficients of each treatment course were *λt*1 = 0.46, *λt*2 = 0.25, and *λt*3 = 0.29.

The state transfer matrix of a 66-year female patient (patient b) in each treatment course was shown as follows:

(18) $$\begin{array}{c} Q\left(t1\right)=\left[\begin{array}{ccccc} 0 & 0 & 0 & 0 & 0\\ 1 & 0 & 0 & 0 & 0\\ 0 & 1 & 0 & 0 & 0\\ 0 & 0 & 1 & 0 & 0\\ 0 & 0 & 0.48 & 0.52 & 0 \end{array}\right],\\ Q\left(t2\right)=\left[\begin{array}{ccccc} 1 & 0 & 0 & 0 & 0\\ 0.71 & 0.29 & 0 & 0 & 0\\ 0 & 1 & 0 & 0 & 0\\ 0 & 0 & 0 & 1 & 0\\ 0 & 0 & 0 & 0 & 0 \end{array}\right],\\ Q\left(t3\right)=\left[\begin{array}{ccccc} 0.76 & 0.24 & 0 & 0 & 0\\ 0.39 & 0.61 & 0 & 0 & 0\\ 0 & 1 & 0 & 0 & 0\\ 0 & 0 & 0 & 1 & 0\\ 0 & 0 & 0 & 0 & 0 \end{array}\right]. \end{array}$$

Her higher-order coefficients of each treatment course were *λt*1 = 0.39, *λt*2 = 0.24, and *λt*3 = 0.37.

The state transfer matrix of a 66-year male patient (patient c) in each treatment course was as follows:

(19) $$\begin{array}{c} Q\left(t1\right)=\left[\begin{array}{ccccc} 0 & 0 & 0 & 0 & 0\\ 1 & 0 & 0 & 0 & 0\\ 0 & 1 & 0 & 0 & 0\\ 0 & 0 & 1 & 0 & 0\\ 0 & 0 & 0 & 1 & 0 \end{array}\right],\\ Q\left(t2\right)=\left[\begin{array}{ccccc} 1 & 0 & 0 & 0 & 0\\ 0 & 1 & 0 & 0 & 0\\ 0 & 0.67 & 0.33 & 0 & 0\\ 0 & 0 & 1 & 0.70 & 0\\ 0 & 0 & 0 & 0 & 0 \end{array}\right],\\ Q\left(t3\right)=\left[\begin{array}{ccccc} 1 & 0 & 0 & 0 & 0\\ 0.40 & 0.60 & 0 & 0 & 0\\ 0 & 0.70 & 0.30 & 0 & 0\\ 0 & 0 & 0 & 0 & 0\\ 0 & 0 & 0 & 0 & 0 \end{array}\right]. \end{array}$$

His higher-order coefficients of each treatment course were *λt*1 = 0.46, *λt*2 = 0.25, and *λt*3 = 0.29.

The state transfer matrix of a 64-year male patient (patient d) in each treatment course was defined as follows:

(20) $$\begin{array}{c} Q\left(t1\right)=\left[\begin{array}{ccccc} 0 & 0 & 0 & 0 & 0\\ 0.55 & 0.45 & 0 & 0 & 0\\ 0.29 & 0.71 & 0 & 0 & 0\\ 0 & 0 & 1 & 0 & 0\\ 0 & 0 & 0.30 & 0.70 & 0 \end{array}\right],\\ Q\left(t2\right)=\left[\begin{array}{ccccc} 1 & 0 & 0 & 0 & 0\\ 0.61 & 0.39 & 0 & 0 & 0\\ 0 & 1 & 0 & 0 & 0\\ 0 & 0 & 0.58 & 0.42 & 0\\ 0 & 0 & 0 & 0 & 0 \end{array}\right],\\ Q\left(t3\right)=\left[\begin{array}{ccccc} 1 & 0 & 0 & 0 & 0\\ 0.26 & 0.74 & 0 & 0 & 0\\ 0 & 1 & 0 & 0 & 0\\ 0 & 0 & 1 & 0 & 0\\ 0 & 0 & 0 & 0 & 0 \end{array}\right], \end{array}$$

His higher-order coefficients of each treatment course were *λt*1 = 0.44, *λt*2 = 0.30, and *λt*3 = 0.26.

The state transfer matrix of a 57-year male patient (patient e) in each treatment course was defined as follows:

(21) $$\begin{array}{c} Q\left(t1\right)=\left[\begin{array}{ccccc} 0 & 0 & 0 & 0 & 0\\ 1 & 0 & 0 & 0 & 0\\ 0 & 1 & 0 & 0 & 0\\ 0 & 0 & 1 & 0 & 0\\ 0 & 0 & 0 & 1 & 0 \end{array}\right],\\ Q\left(t2\right)=\left[\begin{array}{ccccc} 1 & 0 & 0 & 0 & 0\\ 0.56 & 0.44 & 0 & 0 & 0\\ 0 & 0 & 0.63 & 0.37 & 0\\ 0 & 0 & 1 & 0 & 0\\ 0 & 0 & 0 & 0 & 0 \end{array}\right],\\ Q\left(t3\right)=\left[\begin{array}{ccccc} 1 & 0 & 0 & 0 & 0\\ 0.73 & 0.27 & 0 & 0 & 0\\ 0 & 1 & 0 & 0 & 0\\ 0 & 0 & 0 & 0 & 0\\ 0 & 0 & 0 & 0 & 0 \end{array}\right]. \end{array}$$

His higher-order coefficients of each treatment course were *λt*1 = 0.39, *λt*2 = 0.28, and *λt*3 = 0.33.

The state transfer matrix of a 58-year female patient (patient f) in each treatment course was defined as follows:

(22) $$\begin{array}{c} Q\left(t1\right)=\left[\begin{array}{ccccc} 0 & 0 & 0 & 0 & 0\\ 1 & 0 & 0 & 0 & 0\\ 0 & 0.39 & 0.61 & 0 & 0\\ 0 & 0 & 1 & 0 & 0\\ 0 & 0 & 0 & 1 & 0 \end{array}\right],\\ Q\left(t2\right)=\left[\begin{array}{ccccc} 1 & 0 & 0 & 0 & 0\\ 0 & 1 & 0 & 0 & 0\\ 0 & 1 & 0 & 0 & 0\\ 0 & 0 & 1 & 0 & 0\\ 0 & 0 & 0 & 0 & 0 \end{array}\right],\\ Q\left(t3\right)=\left[\begin{array}{ccccc} 1 & 0 & 0 & 0 & 0\\ 0.30 & 0.70 & 0 & 0 & 0\\ 0 & 0.48 & 0.52 & 0 & 0\\ 0 & 0 & 0 & 0 & 0\\ 0 & 0 & 0 & 0 & 0 \end{array}\right]. \end{array}$$

Her higher-order coefficients of each treatment course were *λt*1 = 0.42, *λt*2 = 0.28, and *λt*3 = 0.30.

The state transfer matrix of a 66-year male patient (patient g) in each treatment course was defined as follows:

(23) $$\begin{array}{c} Q\left(t1\right)=\left[\begin{array}{ccccc} 0 & 0 & 0 & 0 & 0\\ 0.5 & 0.5 & 0 & 0 & 0\\ 0 & 0 & 1 & 0 & 0\\ 0 & 0 & 0.79 & 0.21 & 0\\ 0 & 0 & 0.35 & 0.65 & 0 \end{array}\right],\\ Q\left(t2\right)=\left[\begin{array}{ccccc} 1 & 0 & 0 & 0 & 0\\ 1 & 0 & 0 & 0 & 0\\ 0 & 0.56 & 0.23 & 0.21 & 0\\ 0 & 0 & 1 & 0 & 0\\ 0 & 0 & 0 & 0 & 0 \end{array}\right],\\ Q\left(t3\right)=\left[\begin{array}{ccccc} 1 & 0 & 0 & 0 & 0\\ 0.62 & 0.38 & 0 & 0 & 0\\ 0 & 0.65 & 0.35 & 0 & 0\\ 0 & 0 & 1 & 0 & 0\\ 0 & 0 & 0 & 0 & 0 \end{array}\right], \end{array}$$

His higher-order coefficients of each treatment course were *λt*1 = 0.45, *λt*2 = 0.30, and *λt*3 = 0.25.

The state transfer matrix of a 50-year male patient (patient h) in each treatment course was defined as follows:

(24) $$\begin{array}{c} Q\left(t1\right)=\left[\begin{array}{ccccc} 0 & 0 & 0 & 0 & 0\\ 1 & 0 & 0 & 0 & 0\\ 0 & 1 & 0 & 0 & 0\\ 0 & 0.37 & 0.63 & 0 & 0\\ 0 & 0 & 0 & 1 & 0 \end{array}\right],\\ Q\left(t2\right)=\left[\begin{array}{ccccc} 1 & 0 & 0 & 0 & 0\\ 0.66 & 0.34 & 0 & 0 & 0\\ 0 & 1 & 0.58 & 0.42 & 0\\ 0 & 0 & 0.58 & 0.42 & 0\\ 0 & 0 & 0 & 0 & 0 \end{array}\right],\\ Q\left(t3\right)=\left[\begin{array}{ccccc} 1 & 0 & 0 & 0 & 0\\ 1 & 0.70 & 0 & 0 & 0\\ 0 & 0.50 & 0.50 & 0 & 0\\ 0 & 0 & 1 & 0 & 0\\ 0 & 0 & 0 & 0 & 0 \end{array}\right]. \end{array}$$

His higher-order coefficients of each treatment course were *λt*1 = 0.43, *λt*2 = 0.34, and *λt*3 = 0.23.

The state transfer matrix of a 68-year male patient (patient i) in each treatment course was defined as follows:

(25) $$\begin{array}{c} Q\left(t1\right)=\left[\begin{array}{ccccc} 0 & 0 & 0 & 0 & 0\\ 1 & 0 & 0 & 0 & 0\\ 0.48 & 0.52 & 0 & 0 & 0\\ 0 & 0 & 0.79 & 0.21 & 0\\ 0 & 0 & 0.52 & 0.48 & 0 \end{array}\right],\\ Q\left(t2\right)=\left[\begin{array}{ccccc} 0.68 & 0.32 & 0 & 0 & 0\\ 1 & 0 & 0 & 0 & 0\\ 0.29 & 0.45 & 0.26 & 0 & 0\\ 0 & 0 & 0.42 & 0.58 & 0\\ 0 & 0 & 0 & 0 & 0 \end{array}\right],\\ Q\left(t3\right)=\left[\begin{array}{ccccc} 1 & 0 & 0 & 0 & 0\\ 0.71 & 0.29 & 0 & 0 & 0\\ 0 & 1 & 0 & 0 & 0\\ 0 & 0 & 0 & 0 & 0\\ 0 & 0 & 0 & 0 & 0 \end{array}\right]. \end{array}$$

His higher-order coefficients of each treatment course were *λt*1 = 0.36, *λt*2 = 0.30, and *λt*3 = 0.34.

The state transfer matrix of a 61-year female patient (patient j) in each treatment course was defined as follows:

(26) $$\begin{array}{c} Q\left(t1\right)=\left[\begin{array}{ccccc} 0 & 0 & 0 & 0 & 0\\ 0 & 1 & 0 & 0 & 0\\ 0.60 & 0.40 & 0 & 0 & 0\\ 0 & 0 & 1 & 0 & 0\\ 0 & 0 & 0 & 1 & 0 \end{array}\right],\\ Q\left(t2\right)=\left[\begin{array}{ccccc} 0.50 & 0.50 & 0 & 0 & 0\\ 1 & 0 & 0 & 0 & 0\\ 0 & 1 & 0 & 0 & 0\\ 0 & 0.3 & 0.7 & 0 & 0\\ 0 & 0 & 0 & 0 & 0 \end{array}\right],\\ Q\left(t3\right)=\left[\begin{array}{ccccc} 1 & 0 & 0 & 0 & 0\\ 0.51 & 0.49 & 0 & 0 & 0\\ 0 & 0.58 & 0.42 & 0 & 0\\ 0 & 0 & 0 & 0 & 0\\ 0 & 0 & 0 & 0 & 0 \end{array}\right]. \end{array}$$

Her higher-order coefficients of each treatment course were *λt*1 = 0.40, *λt*2 = 0.29, and *λt*3 = 0.31.

The state transfer matrix of a 43-year male patient (patient k) in each treatment course was defined as follows:

(27) $$\begin{array}{c} Q\left(t1\right)=\left[\begin{array}{ccccc} 0 & 0 & 0 & 0 & 0\\ 0.30 & 0.70 & 0 & 0 & 0\\ 0 & 0.67 & 0.33 & 0 & 0\\ 0 & 0 & 1 & 0 & 0\\ 0 & 0 & 0 & 1 & 0 \end{array}\right],\\ Q\left(t2\right)=\left[\begin{array}{ccccc} 1 & 0 & 0 & 0 & 0\\ 0.62 & 0.38 & 0 & 0 & 0\\ 0 & 0.61 & 0.39 & 0 & 0\\ 0 & 0 & 1 & 0 & 0\\ 0 & 0 & 0 & 0 & 0 \end{array}\right],\\ Q\left(t3\right)=\left[\begin{array}{ccccc} 1 & 0 & 0 & 0 & 0\\ 0.79 & 0.21 & 0 & 0 & 0\\ 0 & 1 & 0 & 0 & 0\\ 0 & 0 & 0 & 0 & 0\\ 0 & 0 & 0 & 0 & 0 \end{array}\right]. \end{array}$$

His higher-order coefficients of each treatment course were *λt*1 = 0.40, *λt*2 = 0.24, and *λt*3 = 0.36.

The state transfer matrix of a 58-year male patient (patient l) in each treatment course was defined as follows:

(28) $$\begin{array}{c} Q\left(t1\right)=\left[\begin{array}{ccccc} 0 & 0 & 0 & 0 & 0\\ 0 & 1 & 0 & 0 & 0\\ 0.62 & 0.38 & 0 & 0 & 0\\ 0 & 0 & 0.74 & 0.26 & 0\\ 0 & 0 & 0.27 & 0.73 & 0 \end{array}\right],\\ Q\left(t2\right)=\left[\begin{array}{ccccc} 1 & 0 & 0 & 0 & 0\\ 1 & 0 & 0 & 0 & 0\\ 0 & 0.74 & 0 & 0.26 & 0\\ 0 & 0 & 1 & 0 & 0\\ 0 & 0 & 0 & 0 & 0 \end{array}\right],\\ Q\left(t3\right)=\left[\begin{array}{ccccc} 1 & 0 & 0 & 0 & 0\\ 0.52 & 0.48 & 0 & 0 & 0\\ 0 & 0.63 & 0.37 & 0 & 0\\ 0 & 0 & 1 & 0 & 0\\ 0 & 0 & 0 & 0 & 0 \end{array}\right]. \end{array}$$

His higher-order coefficients of each treatment course were *λt*1 = 0.46, *λt*2 = 0.30, and *λt*3 = 0.24.

The state transfer matrix of a 47-year male patient (patient m) in each treatment course was defined as follows:

(29) $$\begin{array}{c} Q\left(t1\right)=\left[\begin{array}{ccccc} 0 & 0 & 0 & 0 & 0\\ 0 & 0 & 0 & 0 & 0\\ 0.2 & 0.39 & 0.41 & 0 & 0\\ 0 & 0 & 1 & 0 & 0\\ 0 & 0 & 0.45 & 0.55 & 0 \end{array}\right],\\ Q\left(t2\right)=\left[\begin{array}{ccccc} 1 & 0 & 0 & 0 & 0\\ 0.58 & 0.42 & 0 & 0 & 0\\ 0 & 0.82 & 0 & 0.18 & 0\\ 0 & 0 & 1 & 0 & 0\\ 0 & 0 & 0 & 0 & 0 \end{array}\right],\\ Q\left(t3\right)=\left[\begin{array}{ccccc} 1 & 0 & 0 & 0 & 0\\ 0.28 & 0.72 & 0 & 0 & 0\\ 0 & 0 & 1 & 0 & 0\\ 0 & 0 & 1 & 0 & 0\\ 0 & 0 & 0 & 0 & 0 \end{array}\right]. \end{array}$$

His higher-order coefficients of each treatment course were *λt*1 = 0.38, *λt*2 = 0.31, and *λt*3 = 0.31.

The state transfer matrix of a 55-year male patient (patient *n*) in each treatment course was defined as follows:

(30) $$\begin{array}{c} Q\left(t1\right)=\left[\begin{array}{ccccc} 0 & 0 & 0 & 0 & 0\\ 1 & 0 & 0 & 0 & 0\\ 0 & 1 & 0 & 0 & 0\\ 0 & 0 & 0.79 & 0.21 & 0\\ 0 & 0 & 0 & 0 & 0 \end{array}\right],\\ Q\left(t2\right)=\left[\begin{array}{ccccc} 1 & 0 & 0 & 0 & 0\\ 0.24 & 0.76 & 0 & 0 & 0\\ 0 & 0.26 & 0.45 & 0.29 & 0\\ 0 & 0 & 1 & 0 & 0\\ 0 & 0 & 0 & 0 & 0 \end{array}\right],\\ Q\left(t3\right)=\left[\begin{array}{ccccc} 1 & 0 & 0 & 0 & 0\\ 0.73 & 0.28 & 0 & 0 & 0\\ 0 & 0.63 & 0.37 & 0 & 0\\ 0 & 0 & 1 & 0 & 0\\ 0 & 0 & 0 & 0 & 0 \end{array}\right]. \end{array}$$

His higher-order coefficients of each treatment course were *λt*1 = 0.44, *λt*2 = 0.36, and *λt*3 = 0.20.

The state transfer matrix of a 69-year female patient (patient o) in each treatment course was defined as follows:

(31) $$\begin{array}{c} Q\left(t1\right)=\left[\begin{array}{ccccc} 0 & 0 & 0 & 0 & 0\\ 1 & 0 & 0 & 0 & 0\\ 0 & 0.32 & 0.68 & 0 & 0\\ 0 & 0 & 0.70 & 0.30 & 0\\ 0 & 0 & 0 & 1 & 0 \end{array}\right],\\ Q\left(t2\right)=\left[\begin{array}{ccccc} 1 & 0 & 0 & 0 & 0\\ 0 & 1 & 0 & 0 & 0\\ 0 & 0.83 & 0.17 & 0 & 0\\ 0 & 0 & 0.48 & 0.52 & 0\\ 0 & 0 & 0 & 0 & 0 \end{array}\right],\\ Q\left(t3\right)=\left[\begin{array}{ccccc} 1 & 0 & 0 & 0 & 0\\ 0.58 & 0.42 & 0 & 0 & 0\\ 0 & 1 & 0 & 0 & 0\\ 0 & 0 & 1 & 0 & 0\\ 0 & 0 & 0 & 0 & 0 \end{array}\right]. \end{array}$$

Her higher-order coefficients of each treatment course were *λt*1 = 0.43, *λt*2 = 0.32, and *λt*3 = 0.25.

The state transition matrix and the higher-order coefficient were substituted into equation (16) to obtain the predicted CD at time *t*4 (Table 8). In order to verify the accuracy of the prediction of the higher-order Markov chain-SPA model, the traditional first-order Markov chain-SPA model [11] was used to predict the connection degree of *t*4 (Table 8).

**Figure 3:**
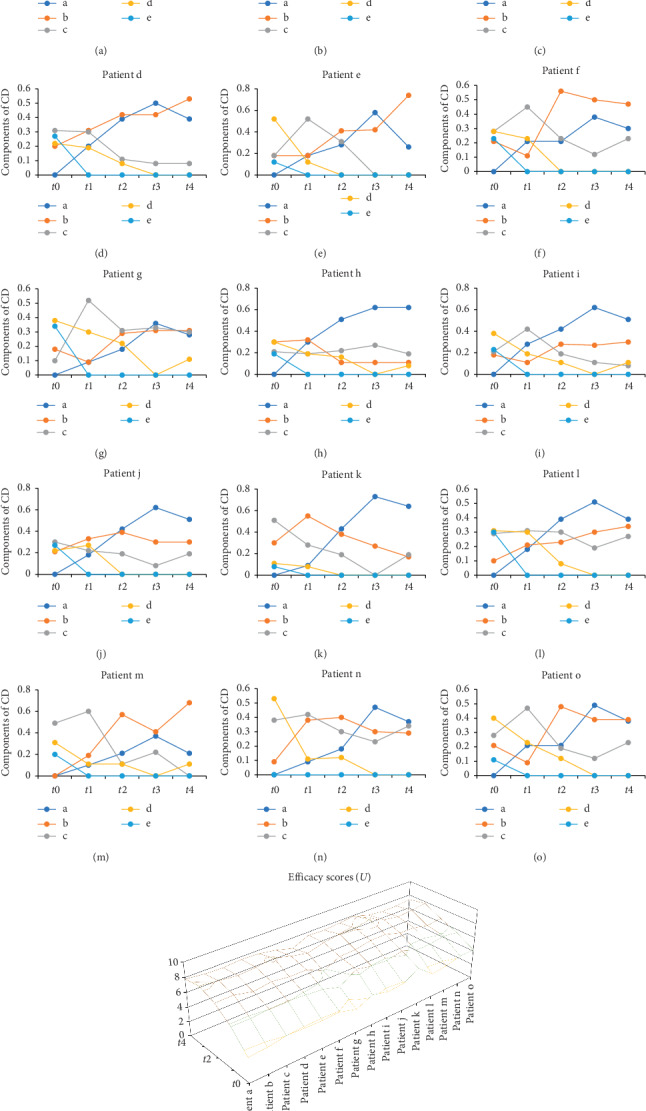
The component of CD of each symptom level and the efficacy score (*U*) trend. (a–o) The connection degrees of each index level of patients changing with time. Legends a–e in the plot represent asymptomatic (I), mild (II), moderate (III), heavier (IV), and severe (V), respectively. (p) The changing trend of curative efficacy score of above 15 patients.

The CD of 15 patients at each time (Table 6) was used to compare the efficacy of each patient in order to better evaluate the curative effect. At the time *t*4, the corresponding CD of 15 patients were *μ* (*t*4) = 0.39 + 0.61*i*, *μ* (*t*4) = 0.26 + 0.74*i*, *μ* (*t*4) = 0.30 + 0.62*i* + 0.08*j*, *μ* (*t*4) = 0.39 + 0.53*i* + 0.08*j*, *μ* (*t*4) = 0.26 + 0.74*i* and *μ* (*t*4) = 0.30 + 0.47*i* + 0.23*j*, *μ* (*t*4) = 0.28 + 0.31*i* + 0.3*j* + 0.11*k*, *μ* (*t*4) = 0.62 + 0.11*i* + 0.19*j* + 0.08*k*, *μ* (*t*4) = 0.51 + 0.30*i* + 0.08*j* + 0.11*k*, *μ* (*t*4) = 0.51 + 0.30*i* + 0.19*j*, *μ* (*t*4) = 0.64 + 0.17*i* + 0.19*j*, *μ* (*t*4) = 0.39 + 0.34*i* + 0.27*j*, *μ* (*t*4) = 0.21 + 0.68*i* + 0.11*k*, *μ* (*t*4) = 0.37 + 0.29*i* + 0.34*j*, and *μ* (*t*4) = 0.38 + 0.39*i* + 0.23*j*, respectively. According to the maximum connection degree principle, the levels of curative effect of 15 patients were I and II. In the *t*3-*t*4 treatment course, there were no significant changes and even slight reduction of calculation results of effect scores (*U*), which proposed that the healing of diabetic ulcer has a certain difficulty. However, the overall curative efficacy score of 15 patients tended to increase (Figure 3), showing that the treatment was therapeutic and the condition has been improved.

According to Table 8, the connection numbers of time *t*4 predicted by the higher-order Markov chain-SPA model were *μ*′ (*t*4) = 0.44 + 0.38*i* + 0.11*j* + 0.08*k*, *μ*′ (*t*4) = 0.30 + 0.46*i* + 0.19*j* + 0.04*k*, *μ*′ (*t*4) = 0.30 + 0.42*i* + 0.25*j* + 0.03*k*, *μ*′ (*t*4) = 0.39 + 0.39*i* + 0.15*j* + 0.07*k*, *μ*′ (*t*4) = 0.36 + 0.34*i* + 0.26*j* + 0.04*k* and *μ*′ (t4) = 0.28 + 0.40*i* + 0.25*j* + 0.07*k*, *μ*′ (*t*4) = 0.24 + 0.25*i* + 0.37*j* + 0.14*k*, *μ*′ (*t*4) = 0.51 + 0.16*i* + 0.23*j* + 0.10*k*, *μ*′ (*t*4) = 0.45 + 0.22*i* + 0.24*j* + 0.10*k*, *μ*′ (*t*4) = 0.42 + 0.34*i* + 0.16*j* + 0.08*k*, *μ*′ (*t*4) = 0.43 + 0.40*i* + 0.15*j* + 0.03*k*, *μ*′ (*t*4) = 0.40 + 0.26*i* + 0.25*j* + 0.09*k*, *μ*′ (*t*4) = 0.24 + 0.39*i* + 0.30*j* + 0.07*k*, *μ*′ (*t*4) = 0.29 + 0.35*i* + 0.29*j* + 0.07*k*, and *μ*′ (*t*4) = 0.33 + 0.34*i* + 0.23*j* + 0.10*k*. The curative efficacy scores were 7.34, 7.03, 6.96, 7.20, 7.06, 6.78, 6.17, 7.16, 7.03, 7.20, 7.46, 6.91, 6.60, 6.73, and 6.83, respectively, and the actual efficacy scores were 7.78, 7.52, 7.44, 7.62, 7.52, 7.14, 6.52, 7.54, 7.42, 7.64, 7.90, 7.24, 6.98, 7.06, and 7.30. The corresponding relative errors of prediction were 5.99%, 6.97%, 6.90%, 5.83%, 6.52%, 5.15%, 5.33%, 5.03%, 5.27%, 5.72%, 5.61%, 4.53%, 5.44%, 4.71%, and 6.51%, indicating that the higher-order Markov chain-SPA curative effect prediction method is effective and can be applied to clinical practice. The connection degrees of time t4 predicted by the first-order Markov chain-SPA model were *μ*^″^ (*t*4) = 0.38 + 0.36*i* + 0.05*j* + 0.02*k* + 0.19*l*, *μ*^″^ (*t*4) = 0.45 + 0.33*i* + 0.07*j* + 0.01*k* + 0.14*l*, *μ*^″^ (*t*4) = 0.45 + 0.33*i* + 0.07*j* + 0.01*k* + 0.14*l*, *μ*^″^ (*t*4) = 0.38 + 0.39*i* + 0.04*j* + 0.02*k* + 0.17*l*, *μ*^″^ (*t*4) = 0.42 + 0.34*i* + 0.09*j* + 0.01*k* + 0.14*l*, *μ*^″^ (*t*4) = 0.32 + 0.40*i* + 0.12*j* + 0.02*k* + 0.19*l*, *μ*^″^ (*t*4) = 0.29 + 0.19*i* + 0.34*j* + 0.08*k* + 0.11*l*, *μ*^″^ (*t*4) = 0.44 + 0.15*i* + 0.19*j* + 0.06*k* + 0.16*l*, *μ*^″^ (*t*4) = 0.46 + 0.19*i* + 0.14*j* + 0.05*k* + 0.15*l*, *μ*^″^ (*t*4) = 0.37 + 0.35*i* + 0.08*j* + 0.02*k* + 0.18*l*, *μ*^″^ (*t*4) = 0.44 + 0.32*i* + 0.08*j* + 0.01*k* + 0.15*l*, *μ*^″^ (*t*4) = 0.37 + 0.28*i* + 0.16*j* + 0.05*k* + 0.14*l*, *μ*^″^ (*t*4) = 0.24 + 0.25*i* + 0.27*j* + 0.02*k* + 0.21*l*, *μ*^″^ (*t*4) = 0.34 + 0.31*i* + 0.23*j* + 0.04*k* + 0.07*l*, and *μ*^″^ (*t*4) = 0.34 + 0.33*i* + 0.18*j* + 0.05*k* + 0.10*l*. The curative efficacy scores were 6.44, 6.91, 6.88, 6.58, 6.78, 6.53, 5.97, 6.29, 6.53, 6.39, 6.80, 6.39, 5.60, 6.60, and 6.51, and the corresponding relative errors were 17.22%, 8.11%, 7.53%, 13.65%, 9.84%, 8.54% 8.50%, 16.62%, 12.01%, 16.35%, 13.89%, 11.78%, 19.79%, 6.53%, and 10.80%. The results of the *t*-test analysis showed that data were normally distributed within the difference between two groups at the significant level of 0.05. The paired *t*-test resulted in *p* < 0.05; the difference was statistically significant (Table 9). We could draw the conclusion that there was a difference on relative error between the groups of high-order Markov chain-SPA and the traditional Markov chain-SPA model (*p* < 0.05), where the high-order Markov chain-SPA curative effect prediction model is more accurate than the first-order Markov chain-SPA model.

## 4. Discussion

Medical decision-making composed of curative effect analysis and prediction is of significance for clinical research. It should be noted that studies mainly put emphasis on evaluating the current curative effect that pays less attention to predicting the change and trend of future curative effect. Based on Markov chain and SPA method, this paper applied a higher-order Markov chain-SPA model for the study of curative effect prediction. At present, SPA and the Markov chain were used in many fields of evaluation and prediction research. For instance, study reported that Bao and Zhang [29] adopted SPA to evaluate the emergency response capacity of large airports so that factors and mechanism of airport vulnerability and emergency response capacity on airport flexibility have been made sure of to improve the resilience of airports. Wang et al. [25] used rank set pair analysis (RSPA) combined with wavelet denoising (WD) to establish hydrometeorological time series prediction model for disaster reduction of drought and flood. The model was compared with the method of conventional Autoregressive Integrated Moving Average (ARIMA), Artificial Neural Networks (ANNs), and RSPA alone where the error of WD-RSPA model was relatively less. Kelly et al. [30] constructed a Markov chain/cellular automata (CA-Markov) model for predicting land use/land cover changes in environments predisposed to desertification. It was found that CA-Markov model could effectively estimate the total land area most easily affected by desertification process. Du et al. [31] proposed a continuous time series Markov model (CTS-MM) for real-time position prediction. The results showed that the position prediction effect of CTS-MM in accurate minutes is better compared with the traditional position prediction model.

The SPA combined with Markov chain model has also been widely applied to prediction analysis successfully in many fields. For instance, Xie and Guo [11] used SPA combined with first-order Markov chain to analyze the influence of human factors in the production process in order to facilitate the management of human factors and reduce the risk of human factors in the production process. It was proved that the prediction model can be used to evaluate the influence of human factors in the actual production process. In the field of information security, Zhang et al. [12] applied SPA and first-order Markov chain to establish a smart grid information security risk assessment model. Compared with the previous smart grid information security risk assessment system, the influence of subjective factors was reduced and every link between the components of the information system was taken into account. In the field of geological engineering, Liu et al. [32] used SPA theory combined with fuzzy-Markov theory to predict the uncertainty coefficient in landslide SPA model. A new SPA-fuzzy-Markov prediction model for landslide deformation was proposed. Compared with SPA model, the composite model was determined to improve the overall prediction accuracy and was of value in practical geotechnical monitoring and analysis. In the field of ecological environment, Qu et al. [33] constructed an eco-impact index. The improved SPA method was used to evaluate the ecological level of Changhe watershed; moreover, Markov chain theory was used to forecast the ecological evolution of the watershed in 2020. This research provided references for the study of ecological evolution of small watersheds.

Although Markov chain has been successfully used to predict the natural progress of diseases in the field of medicine such as the progress of retinopathy in patients with type 2 diabetes mellitus [34], currently there is no literature on the application of Markov chain combined with SPA in medical efficacy evaluation and predictive analysis.

Refractory wound is the most common skin complication of diabetes and the diabetic ulcers are the main cause of nontraumatic amputation of lower extremities. Epidemiological studies have shown that the amputation rate of diabetic feet in countries with a high incidence of diabetes, such as China, is as high as 19.03% [35]. TCM is effective in the treatment of diabetic ulcers, which has the advantages of less scar formation, “nonoperation,” lower cost, and good patient compliance [5, 6]. SJHY treatment has been applied widely in the treatment of chronic refractory ulcers. SPA-CM evaluation model was constructed for evaluating the curative effect of SJHY method in the treatment of diabetic ulcers [8]. On this basis, the establishment of curative effect prediction model is the key that we must pay attention to at present.

This paper suggested a higher-order Markov chain-SPA curative effect prediction model to evaluate the clinical curative effect and predicted the future curative effect through the curative effect state of previous several times. Clinical efficacy always changes with individual differences, environmental factors, and so on. Through the evaluation of clinical curative effect by establishing the SPA model, the CD is composed of five symptom grades and the proportion of the five grades can be clearly presented. The curative efficacy score (*U*) is the reflection of the therapeutic effect. The level of efficacy of patients can be clearly shown. The Markov chain is a commonly used method to describe dynamic random phenomena that predict the future development of the system according to transition probability.

With regard to the weight of symptom indexes of curative effect evaluation, CM was applied [8]. Obviously, applicability of the prediction model plays a core role in affecting the accuracy of prediction. The traditional first-order Markov chain abandons the long-term useful information when describing random phenomena, which makes it easy to bring out the distortion of prediction results in practical application. The curative effect is a sequential process and the previous curative effect also has an impact on the current symptoms. Therefore, this paper extended the traditional first-order Markov chain to higher-order Markov chain for the sake of constructing a curative effect prediction model. In the case, curative effect of the fourth course of treatment was predicted by calculating the curative effect of the first three courses of treatment. The relative errors between the curative efficacy score predicted by the higher-order model and the actual value were 5.99%, 6.97%, 6.90%, 5.83%, 6.52%, 5.15%, 5.33%, 5.03%, 5.27%, 5.72%, 5.61%, 4.53%, 5.44%, 4.71%, and 6.51%, respectively. However, the relative errors of the traditional first-order model were 17.22%, 8.11%, 7.53%, 13.65%, 9.84%, 8.54%, 8.50%, 16.62%, 12.01%, 16.35%, 13.89%, 11.78%, 19.79%, 6.53%, and 10.80% correspondingly, which proves that the higher-order model can be applied to practice and has high accuracy. It provides a new way to predict the curative effect of diabetic ulcer and helps assist clinicians or researchers in making better clinical decisions or evaluating clinical research programs.

## 5. Conclusions

To conclude, the study introduced a curative effect prediction model based on Markov chain and SPA. In this paper, SJHY treatment was applied to treat diabetic ulcer and the curative effect of the first three courses treatment was evaluated to predict the curative effect of the fourth treatment course. The predicted values were compared with the actual values and the predicted values of the traditional first-order model. The following conclusions are obtained:

1. This paper introduced a curative effect prediction model, which used the transfer probability and five-element connection degree between each symptom level to construct the higher-order Markov chain-SPA curative effect prediction model. The model was applied to predict the curative effect of diabetic ulcer. The relative error between the predicted results and the actual value is about 5.70%, indicating that the higher-order efficacy prediction model can be used in clinical efficacy prediction of diabetic ulcers.
2. In this paper, CM was used to calculate the weight of curative effect indexes because of the different importance of different symptoms in the evaluation of curative effect. Due to the limitation of the first-order Markov chain only related to the current and the developed curative effect related to many states after treatment, the traditional first-order Markov chain is extended to the higher-order Markov chain. The results show that this method improves the accuracy of the prediction model.
3. In this paper, CM was used to calculate the constant weight, which reflects the relative importance of the index. The relative error between the prediction result and the actual value is approximately 5.70%, which is far lower than traditional first-order Markov chain. In the future, variable weight method will be used to make the index weight change with the state of the curative effect of the index, which reflects the importance of the state order of the symptom index. Moreover, we will evaluate and predict the curative effect for more and longer time in order to reduce the prediction error and make the prediction more accurate.

## Figures and Tables

**Table 1:** Scores of each index level.

Index levels	Asymptomatic (I)	Lighter (II)	Moderate (III)	Heavier (IV)	Severe (V)
Score	9	7	5	3	1

The value corresponding to each index level is used to calculate the curative effect score. The larger the value is, the better the curative effect is.

**Table 2:** Experts' judgment on importance of different symptoms according to the importance of each symptom.

Linguistic variables level	Value range
Very important	(8,10]
Important	(6,8]
Semi-important	(4,6]
Unimportant	(2,4]
Very unimportant	(0,2]

The experts evaluated the value range of the symptom weight. The larger the score is, the more important the corresponding symptom index is in the curative effect evaluation.

**Table 3:** Classification of grade according to severity score for diabetic ulcers.

Index	Grade				
	I	II	III	IV	V
Wound area (K1: cm^2^)	0-1	1–4	4–9	9–16	>16
Wound depth (K2: cm)	0-1	1-2	2-3	3-4	>4
Exudates color (K3)	Transparent	Red	Yellow	Green	Black
Exudates volume (K4: layers of gauze wetted)	0–4	5–8	9–12	13–16	>16
Necrotic tissue area (K5: %)	0–20	21–40	41–60	61–80	81–100
New granulation and epithelial tissue color (K6)	Bright red	Red	Light red	Pink	Pale
New granulation and epithelial tissue area (K7: %)	81–100	61–80	41–60	21–40	0–20
Wound skin temperature (K8)	Normal	Slightly hot	Hot	Pretty hot	Scorching hot
Wound skin color (K9)	Normal	Reddish	Red	Bright red	Dark red
Pain (K10: VAS)	0–2	3-4	5-6	7-8	9-10

**Table 4:** Index levels at each time point of 15 patients.

Index	*t*_0_	*t*_1_	*t*_2_	*t*_3_	*t*_4_	*t*_0_	*t*_1_	*t*_2_	*t*_3_	*t*_4_	*t*_0_	*t*_1_	*t*_2_	*t*_3_	*t*_4_
	Patient a					Patient b					Patient c				
K1	IV	III	II	II	II	IV	III	II	II	II	III	II	II	II	II
K2	IV	II	II	I	II	V	IV	III	II	II	IV	III	II	I	II
K3	III	II	I	I	I	IV	III	II	II	II	II	I	I	I	I
K4	III	I	I	I	I	II	I	I	I	I	IV	III	II	II	II
K5	II	II	I	I	I	III	II	II	I	II	II	I	I	I	I
K6	IV	III	II	I	II	IV	III	II	I	II	IV	III	II	II	II
K7	V	IV	III	II	II	V	III	II	II	II	V	IV	III	II	II
K8	III	I	I	I	I	III	II	I	I	I	III	II	II	I	I
K9	V	IV	III	II	II	III	II	I	II	II	IV	III	III	III	III
K10	V	IV	II	II	II	III	II	I	I	I	IV	III	III	II	II

	Patient d					Patient e					Patient f				
K1	IV	III	II	II	II	IV	III	II	II	II	III	II	II	II	II
K2	III	II	II	II	II	V	IV	III	II	II	V	IV	III	III	III
K3	II	I	I	I	I	IV	III	II	I	II	II	I	I	I	I
K4	II	II	I	I	I	II	I	I	I	I	IV	III	II	II	II
K5	III	II	I	I	I	III	II	I	I	II	II	I	I	I	I
K6	IV	III	II	I	II	IV	III	II	I	II	IV	III	II	II	II
K7	V	IV	III	II	II	IV	III	III	II	II	V	IV	III	II	III
K8	III	I	I	I	I	II	I	I	I	I	III	III	II	I	I
K9	V	III	II	II	II	IV	III	III	II	II	IV	III	II	I	II
K10	V	IV	IV	III	III	III	II	II	I	I	III	III	II	II	II

	Patient g					Patient h					Patient i				
K1	V	IV	III	III	III	IV	II	II	I	I	IV	III	II	I	II
K2	V	III	III	II	II	III	II	I	I	I	V	III	I	I	I
K3	IV	III	II	II	II	II	I	I	I	I	III	II	I	I	I
K4	II	II	I	I	I	III	II	I	I	I	II	I	II	I	I
K5	III	III	II	I	I	II	I	I	I	I	III	I	I	I	I
K6	IV	III	IV	III	III	V	IV	III	III	III	IV	III	III	II	II
K7	V	IV	IV	III	IV	IV	III	III	II	II	V	IV	IV	III	IV
K8	II	I	I	I	I	II	I	I	I	I	II	I	I	I	I
K9	IV	IV	III	II	III	IV	III	IV	III	III	IV	IV	III	II	III
K10	IV	III	II	I	II	V	IV	IV	III	IV	IV	III	II	II	II

	Patient j					Patient k					Patient l				
K1	IV	III	II	II	II	III	II	I	I	I	V	IV	III	III	III
K2	III	II	I	I	I	III	II	I	I	I	IV	III	II	I	II
K3	II	II	I	I	I	II	II	II	I	I	III	II	I	I	I
K4	III	I	I	I	I	II	I	I	I	I	III	I	I	I	I
K5	II	II	I	I	I	II	II	II	I	I	II	II	I	I	I
K6	IV	III	II	I	II	III	II	I	I	I	IV	III	II	II	II
K7	V	IV	III	II	III	IV	III	III	II	III	V	IV	III	II	II
K8	III	I	II	I	I	III	III	II	I	II	III	I	I	I	I
K9	V	IV	III	III	III	V	IV	III	II	III	V	III	IV	III	III
K10	V	IV	II	II	II	III	III	II	II	II	IV	IV	III	II	III

	Patient m					Patient n					Patient o				
K1	IV	III	II	II	II	III	II	II	I	I	III	III	II	II	II
K2	III	III	II	II	II	IV	III	IV	III	III	IV	IV	IV	III	III
K3	III	II	I	I	I	IV	IV	III	III	III	II	I	I	I	I
K4	IV	III	II	II	II	II	I	I	I	I	IV	III	II	II	I
K5	III	I	I	I	I	III	II	II	I	II	II	I	I	I	I
K6	V	IV	III	III	IV	IV	III	III	II	III	IV	III	II	I	II
K7	IV	III	IV	III	II	IV	III	II	II	II	V	IV	III	II	III
K8	V	III	II	II	II	III	II	I	I	I	III	II	II	I	II
K9	III	II	II	I	II	IV	III	III	II	II	IV	III	III	II	II
K10	III	III	II	I	II	III	II	II	I	I	III	III	II	I	I

The symptom index levels of patients a–o at five time points were obtained by evaluation after every treatment course in the study. Levels I, II, III, IV, and V were asymptomatic, lighter, moderate, heavier, and severe, respectively.

**Table 5:** The CM characteristic numbers and the cloud weight standardized value of each index.

Index	Ex	En	He
Wound area (K1: cm^2^)	0.11073	0.0217	0.00486
Wound depth (K2: cm)	0.12116	0.01886	0.00515
Exudates color (K3)	0.10537	0.01384	0.00412
Exudates volume (K4: layers of gauze wetted)	0.0891	0.01365	0.00451
Necrotic tissue area (K5: %)	0.09907	0.01335	0.00348
New granulation and epithelial tissue color (K6)	0.10844	0.01164	0.00255
New granulation and epithelial tissue area (K7: %)	0.10733	0.01166	0.00280
Wound skin temperature (K8)	0.09309	0.01464	0.00168
Wound skin color (K9)	0.08363	0.01205	0.00308
Pain (K10: VAS)	0.08207	0.01385	0.00154

Ex, En, and He were the CM characteristic numbers corresponding to each symptom index *k*, which were used to calculate cloud weights. Ex, En, and He denote the expected value, entropy, and hyperentropy, respectively. Ex is the expected value of the cloud drop which can represent the qualitative concept. En reflects the dispersion degree of cloud drops, which also determines the certainty of cloud drops. He is the entropy of En and reveals the uncertainty measurement of En which is used to settle confusion degree. *ω* is defined as the standardized value of cloud weight.

**Table 6:** Calculation results of CD.

Patient	Time	I	II	III	IV	V
Patient a	*t*0	0	0.1	0.29	0.34	0.27
	*t*1	0.18	0.33	0.22	0.27	0
	*t*2	0.39	0.42	0.19	0	0
	*t*3	0.62	0.38	0	0	0
	*t*4	0.39	0.61	0	0	0

Patient b	*t*0	0	0.09	0.35	0.33	0.23
	*t*1	0.09	0.35	0.44	0.12	0
	*t*2	0.34	0.54	0.12	0	0
	*t*3	0.47	0.53	0	0	0
	*t*4	0.26	0.74	0	0	0

Patient c	*t*0	0	0.21	0.2	0.48	0.11
	*t*1	0.21	0.2	0.48	0.11	0
	*t*2	0.21	0.52	0.27	0	0
	*t*3	0.42	0.5	0.08	0	0
	*t*4	0.3	0.62	0.08	0	0

Patient d	*t*0	0	0.2	0.31	0.22	0.27
	*t*1	0.2	0.31	0.3	0.19	0
	*t*2	0.39	0.42	0.11	0.08	0
	*t*3	0.5	0.42	0.08	0	0
	*t*4	0.39	0.53	0.08	0	0

Patient e	*t*0	0	0.18	0.18	0.52	0.12
	*t*1	0.18	0.18	0.52	0.12	0
	*t*2	0.28	0.41	0.31	0	0
	*t*3	0.58	0.42	0	0	0
	*t*4	0.26	0.74	0	0	0

Patient f	*t*0	0	0.21	0.28	0.28	0.23
	*t*1	0.21	0.11	0.45	0.23	0
	*t*2	0.21	0.56	0.23	0	0
	*t*3	0.38	0.5	0.12	0	0
	*t*4	0.3	0.47	0.23	0	0

Patient g	*t*0	0	0.18	0.1	0.38	0.34
	*t*1	0.09	0.09	0.52	0.3	0
	*t*2	0.18	0.29	0.31	0.22	0
	*t*3	0.36	0.31	0.33	0	0
	*t*4	0.28	0.31	0.3	0.11	0

Patient h	*t*0	0	0.3	0.21	0.3	0.19
	*t*1	0.3	0.32	0.19	0.19	0
	*t*2	0.51	0.11	0.22	0.16	0
	*t*3	0.62	0.11	0.27	0	0
	*t*4	0.62	0.11	0.19	0.08	0

Patient i	*t*0	0	0.18	0.21	0.38	0.23
	*t*1	0.28	0.11	0.42	0.19	0
	*t*2	0.42	0.28	0.19	0.11	0
	*t*3	0.62	0.27	0.11	0	0
	*t*4	0.51	0.3	0.08	0.11	0

Patient j	*t*0	0	0.21	0.3	0.22	0.27
	*t*1	0.18	0.33	0.22	0.27	0
	*t*2	0.42	0.39	0.19	0	0
	*t*3	0.62	0.3	0.08	0	0
	*t*4	0.51	0.3	0.19	0	0

Patient k	*t*0	0	0.3	0.51	0.11	0.08
	*t*1	0.09	0.55	0.28	0.08	0
	*t*2	0.43	0.38	0.19	0	0
	*t*3	0.73	0.27	0	0	0
	*t*4	0.64	0.17	0.19	0	0

Patient l	*t*0	0	0.1	0.29	0.31	0.3
	*t*1	0.18	0.21	0.31	0.3	0
	*t*2	0.39	0.23	0.3	0.08	0
	*t*3	0.51	0.3	0.19	0	0
	*t*4	0.39	0.34	0.27	0	0

Patient m	*t*0	0	0	0.49	0.31	0.2
	*t*1	0.1	0.19	0.6	0.11	0
	*t*2	0.21	0.57	0.11	0.11	0
	*t*3	0.37	0.41	0.22	0	0
	*t*4	0.21	0.68	0	0.11	0

Patient n	*t*0	0	0.09	0.38	0.53	0
	*t*1	0.09	0.38	0.42	0.11	0
	*t*2	0.18	0.4	0.3	0.12	0
	*t*3	0.47	0.3	0.23	0	0
	*t*4	0.37	0.29	0.34	0	0

Patient o	*t*0	0	0.21	0.28	0.4	0.11
	*t*1	0.21	0.09	0.47	0.23	0
	*t*2	0.21	0.48	0.19	0.12	0
	*t*3	0.49	0.39	0.12	0	0
	*t*4	0.38	0.39	0.23	0	0

The connection degree components of each index level of patients a–o were listed separately at the five time points in the study. I, II, III, IV, and V represent five index levels of asymptomatic, lighter, moderate, heavier, and severe, respectively.

**Table 7:** Calculation results of efficacy scores (*U*).

Patient	*t*0	*t*1	*t*2	*t*3	*t*4
Patient a	3.44	5.84	7.4	8.24	7.78
Patient b	3.6	5.82	7.44	7.94	7.52
Patient c	4.02	6.02	6.88	7.68	7.44
Patient d	3.88	6.04	7.24	7.84	7.62
Patient e	3.84	5.84	6.94	8.16	7.52
Patient f	3.94	5.6	6.96	7.52	7.14
Patient g	3.24	4.94	5.86	7.06	6.52
Patient h	4.24	6.46	6.94	7.7	7.54
Patient i	3.68	5.96	7.02	8.02	7.42
Patient j	3.9	5.84	7.46	8.08	7.64
Patient k	5.06	6.3	7.48	8.46	7.9
Patient l	3.38	5.54	6.86	7.64	7.24
Patient m	3.58	5.56	6.76	7.3	6.98
Patient n	4.12	5.9	6.28	7.48	7.06
Patient o	4.18	5.56	6.56	7.74	7.3

The efficacy scores of patients a–o at each time point were calculated by using equation (5) in the study.

**Table 8:** The connection degree (CD) of predicted and actual and corresponding efficacy scores (*U*).

Patient	Results	The five-element connection degree	Efficacy scores (*U*)	Relative error (%)
Patient a	Actual results	*μ* (*t*4) = 0.39 + 0.61*i*	7.78	
	Predicted results of higher-order Markov chain-SPA	*μ*′ (*t*4) = 0.44 + 0.38*i* + 0.11*j* + 0.08*k*	7.34	5.99
	Predicted results of traditional Markov chain-SPA	*μ* ^″^ (*t*4) = 0.38 + 0.36*i* + 0.05*j* + 0.02*k* + 0.19*l*	6.44	17.22

Patient b	Actual results	*μ* (*t*4) = 0.26 + 0.74*i*	7.52	
	Predicted results of higher-order Markov chain-SPA	*μ*′ (*t*4) = 0.30 + 0.46*i* + 0.19*j* + 0.04*k*	7.03	6.97
	Predicted results of traditional Markov chain-SPA	*μ* ^″^ (*t*4) = 0.45 + 0.33*i* + 0.07*j* + 0.01*k* + 0.14*l*	6.91	8.11

Patient c	Actual results	*μ* (*t*4) = 0.30 + 0.62*i* + 0.08*j*	7.44	
	Predicted results of higher-order Markov chain-SPA	*μ*′ (*t*4) = 0.30 + 0.42*i* + 0.25*j* + 0.03*k*	6.96	6.90
	Predicted results of traditional Markov chain-SPA	*μ* ^″^ (*t*4) = 0.45 + 0.33*i* + 0.07*j* +0 .01*k* + 0.14*l*	6.88	7.53

Patient d	Actual results	*μ* (*t*4) = 0.39 + 0.53*i* + 0.08*j*	7.62	
	Predicted results of higher-order Markov chain-SPA	*μ*′ (*t*4) = 0.39 + 0.39*i* + 0.15*j* + 0.07*k*	7.20	5.83
	Predicted results of traditional Markov chain-spa	*μ* ^″^ (*t*4) = 0.38 + 0.39*i* + 0.04*j* + 0.02*k* + 0.17*l*	6.58	13.65

Patient e	Actual results	*μ* (*t*4) = 0.26 + 0.74*i*	7.52	
	Predicted results of higher-order Markov chain-SPA	*μ*′ (*t*4) = 0.36 + 0.34*i* + 0.26*j* + 0.04*k*	7.06	6.52
	Predicted results of traditional Markov chain-SPA	*μ* ^″^ (*t*4) = 0.42 + 0.34*i* + 0.09*j* + 0.01*k* + 0.14*l*	6.78	9.84

Patient f	Actual results	*μ* (*t*4) = 0.30 + 0.47*i* + 0.23*j*	7.14	
	Predicted results of higher-order Markov chain-SPA	*μ*′ (*t*4) = 0.28 + 0.40*i* + 0.25*j* + 0.07*k*	6.78	5.15
	Predicted results of traditional Markov chain-SPA	*μ* ^″^ (*t*4) = 0.32 + 0.40*i* + 0.12*j* + 0.02*k* + 0.19*l*	6.53	8.54

Patient g	Actual results	*μ* (*t*4) = 0.28 + 0.31*i* + 0.3*j* + 0.11*k*	6.52	
	Predicted results of higher-order Markov chain-SPA	*μ*′ (*t*4) = 0.24 + 0.25*i* + 0.37*j* + v0.14*k*	6.17	5.33
	Predicted results of traditional Markov chain-SPA	*μ* ^″^ (*t*4) = 0.29 + 0.19*i* + 0.34*j* + 0.08*k* + 0.11*l*	5.97	8.50

Patient h	Actual results	*μ* (*t*4) = 0.62 + 0.11*i* + 0.19*j* + 0.08*k*	7.54	
	Predicted results of higher-order Markov chain-SPA	*μ*′ (*t*4) = 0.51 + 0.16*i* + 0.23*j* + 0.10*k*	7.16	5.03
	Predicted results of traditional Markov chain-SPA	*μ* ^″^ (*t*4) = 0.44 + 0.15*i* + 0.19*j* + 0.06*k* + 0.16*l*	6.29	16.62

Patient i	Actual results	*μ* (*t*4) = 0.51 + 0.30*i* + 0.08*j* + 0.11*k*	7.42	
	Predicted results of higher-order Markov chain-SPA	*μ*′ (*t*4) = 0.45 + 0.22*i* + 0.24*j* + 0.10*k*	7.03	5.27
	Predicted results of traditional Markov chain-SPA	*μ* ^″^ (*t*4) = 0.46 + 0.19*i* + 0.14*j* + 0.05*k* + 0.15*l*	6.53	12.01

Patient j	Actual results	*μ* (*t*4) = 0.51 + 0.30*i* + 0.19*j*	7.64	
	Predicted results of higher-order Markov chain-SPA	*μ*′ (*t*4) = 0.42 + 0.34*i* + 0.16*j* + 0.08*k*	7.20	5.72
	Predicted results of traditional Markov chain-SPA	*μ* ^″^ (*t*4) = 0.37 + 0.35*i* + 0.08*j* + 0.02*k* + 0.18*l*	6.39	16.35

Patient k	Actual results	*μ* (*t*4) = 0.64 + 0.17*i* + 0.19*j*	7.90	
	Predicted results of higher-order Markov chain-SPA	*μ*′ (*t*4) = 0.43 + 0.40*i* + 0.15*j* + 0.03*k*	7.46	5.61
	Predicted results of traditional Markov chain-SPA	*μ* ^″^ (*t*4) = 0.44 + 0.32*i* + 0.08*j* + 0.01*k* + 0.15*l*	6.80	13.89

Patient l	Actual results	*μ* (*t*4) = 0.39 + 0.34*i* + 0.27*j*	7.24	
	Predicted results of higher-order Markov chain-SPA	*μ*′ (*t*4) = 0.40 + 0.26*i* + 0.25*j* + 0.09*k*	6.91	4.53
	Predicted results of traditional Markov chain-SPA	*μ* ^″^ (*t*4) = 0.37 + 0.28*i* + 0.16*j* + 0.05*k* + 0.14*l*	6.39	11.78

Patient m	Actual results	*μ* (*t*4) = 0.21 + 0.68*i* + 0.11*k*	6.98	
	Predicted results of higher-order Markov chain-SPA	*μ*′ (*t*4) = 0.24 + 0.39*i* + 0.30*j* + 0.07*k*	6.60	5.44
	Predicted results of traditional Markov chain-SPA	*μ* ^″^ (*t*4) = 0.24 + 0.25*i* + 0.27*j* + 0.02*k* + 0.21*l*	5.60	19.79

Patient n	Actual results	*μ* (*t*4) = 0.37 + 0.29*i* + 0.34*j*	7.06	
	Predicted results of higher-order Markov chain-SPA	*μ*′ (*t*4) = 0.29 + 0.35*i* + 0.29*j* + 0.07*k*	6.73	4.71
	Predicted results of traditional Markov chain-SPA	*μ* ^″^ (*t*4) = 0.34 + 0.31*i* + 0.23*j* + 0.04*k* + 0.07*l*	6.60	6.53

Patient o	Actual results	*μ* (*t*4) = 0.38 + 0.39*i* + 0.23*j*	7.30	
	Predicted results of higher-order Markov chain-SPA	*μ*′ (*t*4) = 0.33 + 0.34*i* + 0.23*j* + 0.10*k*	6.83	6.51
	Predicted results of traditional Markov chain-SPA	*μ* ^″^ (*t*4) = 0.34 + 0.33*i* + 0.18*j* + 0.05*k* + 0.10*l*	6.51	10.80

The connection degrees predicted by higher-order Markov chain-SPA model and efficacy scores at time *t*4 were compared with the actual connection degrees and the connection degrees predicted by the traditional first-order Markov chain-SPA model. The relative error is the proportion of the difference between the predicted value and the actual value in the predicted value.

**Table 9:** Comparison of the relative error between two groups of high-order Markov chain-SPA and the traditional Markov chain-SPA model.

Groups	Means ± SD
Higher-order Markov chain-SPA model	0.0570 ± 0.0073^*∗*^
Traditional Markov chain-SPA model	0.1208 ± 0.0392

^*∗*^ *p* < 0.05 compared with the traditional Markov chain-SPA model.

## Data Availability

All of the data used to support the findings of this study are available from the corresponding author upon request.

## Abbreviations

ANNs: Artificial neural networks
ARIMA: Autoregressive integrated moving average
CA-Markov: Markov chain/cellular automata
CD: Connection degree
CM: Cloud model
CTS-MM: Continuous time series Markov model
RSPA: Rank set pair analysis
SJHY: Sheng-ji Hua-yu
SPA: Set pair analysis
TCM: Traditional Chinese medicine
WD: Wavelet denoising.

## References

[1] Wang Y. R., Ren L. F., Sun Z. Q. (2012). Novel dynamic topsis method in evaluation for quality of medical care. *Zhong Nan Da Xue Xue Bao Yi Xue Ban*.

[2] Xu W. F., Gu W. J., Liu G. P. (2016). Study on feature selection and syndrome classification of excess syndrome in chronic gastritis based on random forest algorithm and multi-label learning. *Chinese Journal of Information on Traditional Chinese Medicine*.

[3] Cho N. H., Shaw J. E., Karuranga S. (2018). IDF diabetes atlas: global estimates of diabetes prevalence for 2017 and projections for 2045. *Diabetes Research and Clinical Practice*.

[4] Assaad-Khalil S. H., Zaki A., Rehim A. A. (2015). Prevalence of diabetic foot disorders and related risk factors among Egyptian subjects with diabetes. *Primary Care Diabetes*.

[5] Li F.-L., Deng H., Wang H.-W. (2011). Effects of external application of Chinese medicine on diabetic ulcers and the expressions of *β*-catenin, c-myc and K6. *Chinese Journal of Integrative Medicine*.

[6] Li F. L., Wang Y. F., Li X. (2012). Characteristics and clinical managements of chronic skin ulcers based on traditional chinese medicine. *Evidence-Based Complementary and Alternative Medicine*.

[7] Kuai L., Zhang J. T., Deng Y. (2018). Sheng-ji hua-yu formula promotes diabetic wound healing of re-epithelization via activin/follistatin regulation. *BMC Complementary & Alternative Medicine*.

[8] Kuai L., Xing J. Q., Zhang J. T. (2019). Application of a cloud model-set pair analysis in efficacy assessment for diabetic ulcers. *Evidence-Based Complementary and Alternative Medicine*.

[9] Yang M. J., Zhou S., Meng Q. G. (2018). Construction and exploration of TCM clinical efficacy evaluation based on markov model. *Chinese Archives of Traditional Chinese Medicine*.

[10] Zhao K. Q., Xuan A. L. (1996). Set pair theory-a new theory method of non-define and its applications. *Systems Engineering*.

[11] Xie X., Guo D. (2018). Human factors risk assessment and management: process safety in engineering. *Process Safety and Environmental Protection*.

[12] Zhang J. Y., Zeng Q. S., Song Y. Y., Li C. B. (2014). Information security risk assessment of smart grid based on absorbing markov chain and SPA. *International Journal of Emerging Electric Power Systems*.

[13] Kang Y., Xu K. L., Liu J. X. (2012). SPA-markov chain model for evaluating and forecasting urban buried gas pipeline risk. *China Safety Science*.

[14] Chen S., Shi F. Q., Zhu Z. R., Wu K. (2016). SPA of safety state evolution of hydropower station production environment. *China Safety Science*.

[15] Chowdhury R. I., Islam M. A., Shah M. A., Al-Enezi N. (2005). A computer program to estimate the parameters of covariate dependent higher order markov model. *Computer Methods and Programs in Biomedicine*.

[16] Yang H., Li Y., Lu L., Qi R. (2011). First order multivariate markov chain model for generating annual weather data for Hong Kong. *Energy and Buildings*.

[17] Ching W. K., Fung E. S., Ng M. K. (2003). Higher-order hidden markov models with applications to DNA sequences. *Intelligent Data Engineering and Automated Learning*.

[18] Raftery A. E. (1985). A model for high-order markov chains. *Journal of the Royal Statistical Society: Series B*.

[19] Chang Z. P., Liu X. D., Zhang S. T. (2018). Set pair prediction model for social risk from major decision-making based on variable weight and higher-order markov chain. *Control and Decision*.

[20] Yan F., Xu K. L. (2017). Application of a cloud model-set pair analysis in hazard assessment for biomass gasification stations. *PLoS One*.

[21] Raftery A., Tavare S. (1994). Estimation and modelling repeated patterns in high order markov chains with the mixture transition distribution model. *Applied Statistics*.

[22] Ching W. K., Fung E. S., Ng M. K. (2004). Higher-order markov chain models for categorical data sequences. *Naval Research Logistics*.

[23] Zhai D. H., Li T. L., Duan W. X., Yu J., Xiao J. (2014). Optimal exemplar matching algorithm based on matrix similarity and its application in image inpainting. *Computer Science*.

[24] Wu M. F., Yan L., Guo D. J. (2018). Microbial diversity of chronic wound and successful management of traditional chinese medicine. *Evidence-Based Complementary and Alternative Medicine*.

[25] Wang D., Borthwick A. G., He H. (2018). A hybrid wavelet de-noising and rank-set pair analysis approach for forecasting hydro-meteorological time series. *Environmental Research*.

[26] Kim M., Ghate A., Phillips M. H. (2009). A markov decision process approach to temporal modulation of dose fractions in radiation therapy planning. *Physics in Medicine and Biology*.

[27] Jeffcoate W., Russell D., Boyko E. J. (2020). Guidelines on the classification of diabetic foot ulcers (IWGDF 2019). *Diabetes/Metabolism Research and Reviews*.

[28] Beckert S., Witte M., Wicke C., Konigsrainer A., Coerper S. (2006). A new wound-based severity score for diabetic foot ulcers: a prospective analysis of 1,000 patients. *Diabetes Care*.

[29] Bao D. W., Zhang X. L. (2018). Measurement methods and influencing mechanisms for the resilience of large airports under emergency events. *Transportmetrica A: Transport Science*.

[30] Kelly O., Carlos A., Gustavo E. (2018). Markov chains and cellular automata to predict environments subject to desertification. *Journal of Environmental Management*.

[31] Du Y. P., Wang C. C., Qiao Y. L., Zhao D. Y., Guo W. Y. (2018). A geographical location prediction method based on continuous time series markov model. *PLoS One*.

[32] Liu X., Tang H. M., Liu Y. (2009). A new model for landslide displacement prediction based on set pair analysis and fuzzy-markov chain. *Rock and Soil Mechanics*.

[33] Qu L. L., Shi X. Y., Wang B. Y. (2019). Ecological evolution and impact tracking assessment of the coal-buried villages in the hilly region based on improved SPA-markov: a case study of changhe watershed. *Ecology and Environmental Sciences*.

[34] Srikanth P. (2015). Using markov chains to predict the natural progression of diabetic retinopathy. *International Journal of Ophthalmology*.

[35] Jiang Y., Ran X., Jia L. (2015). Epidemiology of type 2 diabetic foot problems and predictive factors for amputation in China. *The International Journal of Lower Extremity Wounds*.

